# A potent virucidal activity of functionalized TiO_2_ nanoparticles adsorbed with flavonoids against SARS-CoV-2

**DOI:** 10.1007/s00253-022-12112-9

**Published:** 2022-08-11

**Authors:** Gabriela León-Gutiérrez, James Edward Elste, Carlos Cabello-Gutiérrez, Cesar Millán-Pacheco, Mario H. Martínez-Gómez, Rafael Mejía-Alvarez, Vaibhav Tiwari, Armando Mejía

**Affiliations:** 1 Departamento de Biotecnología, Universidad Autónoma Metropolitana–Iztapalapa, Ciudad de Mexico, Mexico; 2 Department of Microbiology and Immunology, Midwestern University, Downers Grove, IL USA; 3 Departamento de Virología e Investigación en Micología, Instituto Nacional de Enfermedades Respiratorias, Ciudad de Mexico, Mexico; 4 Facultad de Farmacia, Universidad Autónoma del Estado de Morelos, Cuernavaca, Morelos, Mexico; 5 Department of Physiology, College of Graduate Studies, Midwestern University, Downers Grove, IL USA

**Keywords:** Nanoparticulate compound, Hesperetin-7-rutinoside, Flavanone-7-O-glucoside, SARS-CoV-2 spike glycoprotein

## Abstract

**Abstract:**

The coronavirus SARS-CoV-2 has caused a pandemic with > 550 millions of cases and > 6 millions of deaths worldwide. Medical management of COVID-19 relies on supportive care as no specific targeted therapies are available yet. Given its devastating effects on the economy and mental health, it is imperative to develop novel antivirals. An ideal candidate will be an agent that blocks the early events of viral attachment and cell entry, thereby preventing viral infection and spread. This work reports functionalized titanium dioxide (TiO_2_)-based nanoparticles adsorbed with flavonoids that block SARS-CoV-2 entry and fusion. Using molecular docking analysis, two flavonoids were chosen for their specific binding to critical regions of the SARS-CoV-2 spike glycoprotein that interacts with the host cell angiotensin-converting enzyme-2 (ACE-2) receptor. These flavonoids were adsorbed onto TiO_2_ functionalized nanoparticles (FTNP). This new nanoparticulate compound was assayed in vitro against two different coronaviruses; HCoV 229E and SARS-CoV-2, in both cases a clear antiviral effect was observed. Furthermore, using a reporter-based cell culture model, a potent antiviral activity is demonstrated. The adsorption of flavonoids to functionalized TiO_2_ nanoparticles induces a ~ threefold increase of that activity. These studies also indicate that FTNP interferes with the SARS-CoV-2 spike, impairing the cell fusion mechanism.

**Key points/Highlights:**

*• Unique TiO*_*2*_ *nanoparticles displaying flavonoid showed potent anti-SARS-CoV-2 activity.*

*• The nanoparticles precisely targeting SARS-CoV-2 were quantitatively verified by cell infectivity in vitro.*

*• Flavonoids on nanoparticles impair the interactions between the spike glycoprotein and ACE-2 receptor.*

**Graphical abstract:**

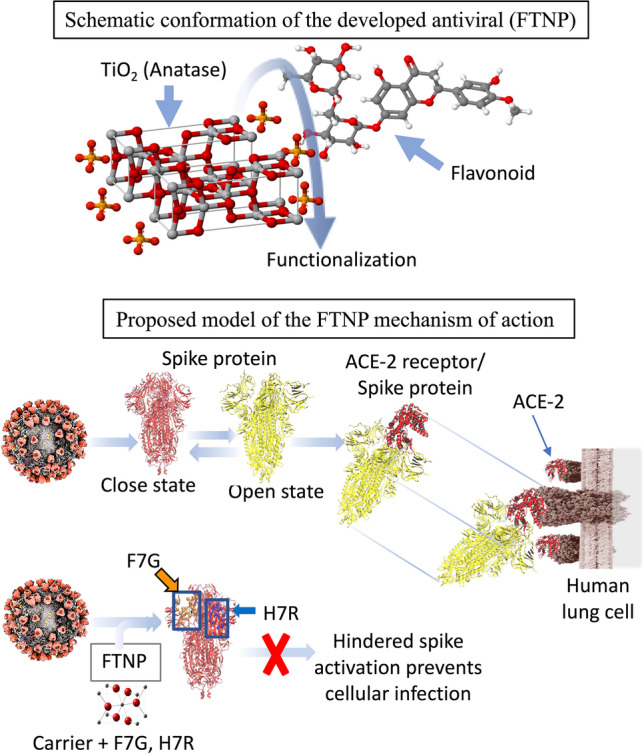

**Supplementary Information:**

The online version contains supplementary material available at 10.1007/s00253-022-12112-9.

## Introduction

Recently, the search for an effective cure for viral diseases like severe acute respiratory syndrome coronavirus (SARS-CoV-2) has dramatically increased. In November 2021, Pfizer developed a combinational antiviral oral medication (PAXLOVID, which inhibits viral replication by targeting the 3CL protease, thus preventing progression to severe COVID-19 (Center for Drug Evaluation 2022). It is important to note that current and other existing drugs do not prevent SARS-CoV-2 entry or cell-to-cell spread which leads to the clinical manifestations. In addition, with the recent emergence of highly pathogenic variants of SARS-CoV-2, there is a genuine concern that multiple new escape 3CL variants could emerge in the future and may lead to severe epidemic rebounds. Although the COVID-19 vaccine has proven vital in in preventing severe disease and controlling the pandemic’s natural history, the development of specific antiviral drugs to treat this disease is still imperative. Since SARS-CoV eradication is highly unlikely, there is an urgent need for an agent that can intervene in the very first steps of interactions between the SARS-CoV-2 and host cell receptor. In this regard, drug repurposing has been a common alternative due to the urgent nature of the problem. A common strategy for selecting potential drugs has been the identification of molecules with the potential to interact with human proteins—presumable viral targets—, to disrupt the infective interaction. This is the case with anticancer drugs (e.g., midostaurin, daunorubicin, ponatinib, silmitasertib), antiepileptic agents (valproic acid), chaperones (miglastat), structural-interaction analogs (melatonin, mercaptopurine, sirolimus, toremifene, emodin, etc. (Zhou et al. 2020)), antiparasitic (ivermectin (Caly et al. 2020), and antimalarial medications (e.g., chloroquine and hydroxychloroquine which supposedly interact with SARS-CoV-2 Sigma receptor (Alexander et al. 2020), alone or in combination with other agents, like antibiotics (i.e., azithromycin, which allegedly shares a common mitochondrial target protein with SARS-CoV-2 (Tyszka et al. 2020). Although certain drugs (like antimalarial) have been highly publicized, their therapeutic benefit has not been demonstrated to be significant. Interestingly, the broad-spectrum antiviral remdesivir (effective against Ebola, Nipah, respiratory syncytial virus family) has shown to be efficacious in shortening the recovery time (from 15 to 10 days) in hospitalized adult patients with severe COVID-19 (Awadhesh et al. 2020). Remdesivir became the first drug to receive emergency use authorization from the U.S. Food & Drug Administration to treat hospitalized patients with COVID-19. However, remdesivir has shown to be effective only in patients with severe disease (i.e., requiring mechanical ventilation, O_2_ saturation < 94% breathing ambient air or respiratory rate ≥ 24 bpm). In contrast, a comprehensive review recently published of repurposed antiviral drugs trials against COVID-19 indicates that hydroxychloroquine, lopinavir, and interferon regimens appeared to have essentially no effect on hospitalized COVID-19 patients, as indicated by overall mortality, initiation of ventilation, and duration of hospital stay (Kausar et al. 2021).

A novel approach with great therapeutic potential is based on the use of nanoparticles (NPs) designed to mimic heparan sulfate proteoglycans (HSPG). HSPG are highly conserved viral attachment ligands (Tiwari et al., 2020); thus, HSPG-based NPs are intended to promote associations with the virus to induce irreversible deformations of its structural proteins reducing its infectivity. Numerous studies ranging from in vitro virucidal assays, electron microscopy imaging, and molecular dynamics simulations support this mechanism of action (Cagno et al. 2019). Furthermore, recent reports indicate that this irreversible antiviral activity of NPs against several types of viruses takes place without inducing cytotoxic effects on the host cell. A robust body of evidence indicates that within therapeutic ranges, the use of TiO_2_ NPs has shown essentially no negative side effects (European Food Safety Authority; EFSA 2019). Reports of ZnO NPs antiviral activity include a variety of viruses like Zika (Fisher and Phillips 2008), herpes simplex type 1 (HSV-1) (Mishra et al., 2011), human papillomavirus, respiratory syncytial, dengue (Cho et al. 2014), influenza H1N1 (Mishra et al. 2011), and avian influenza (Moulick et al. 2017). More recently, non-functionalized TiO_2_ NPs have been successfully used to inactivate the influenza virus, presumably through a similar mechanism of action (Mazurkova et al. 2010). What is even more intriguing is that nanotechnological approaches based on silver have been effectively used against SARS-CoV-2 (Pilaquinga et al. 2021). In addition, functionalized carrying bioactive molecules have been used with high specificity for SARS-CoV-2. However, most of these studies have been aimed at the development of vaccines and viral antigen detection systems (Jamalipour and Iravani 2021).

An alternative biotechnological approach using secondary metabolites like flavonoids and terpenes has recently emerged with great therapeutic potential against viral diseases. This is the case of the flavonoids apigenin, diosmetin, luteolin, acacetin, chrysoeriol, and their respective glycosides, which have been reported to exhibit effective antiviral activities in several diseases (Sharma et al. 2020). Furthermore, the therapeutic potential of some flavonoids has already been reported against SARS-CoV-2 (Table 1). Terpenes have also been found to be active against a wide variety of viruses, particularly against coronaviruses, and with low cytotoxicity as well (Boukhatem 2020). Possible mechanisms of action of flavonoids and terpenoids include inhibition of virus replication by glycosylation of viral proteins and infectivity reduction through interactions with viral envelope lipids (Paduch et al. 2007). Consequently, the specific and wide-ranging antiviral activities of these metabolites could offer a powerful alternative to improve the efficacy of antiviral therapy.

**Table 1:** Significance of flavonoids for generating broad-spectrum targets against SARS-CoV-2

Flavonoids	SARS-CoV-2 target	References
Baicalin, herbacetin, pectolinarin	3 C-like protease (3CLpro)	(Jo et al. 2020)
Quercetin	Antiviral	(Colunga Biancatelli et al. 2020)
Quercetin	Papain-like protease (PLpro)	(Park et al. 2017)
Quercetin and its derivatives	Helicase	(Keum and Jeong 2012)
Cyanidin, delphinidin	ACE-2	(Ojeda et al. 2010)
Epigallocatechin gallate	ACE-2	(Guerrero et al. 2012)
Hesperetin, myricetin	Helicase and protease	(Ngwa et al. 2020)
Catechin gallate	SARS-CoV N protein	(Roh 2012)
Isorhamnetin-3-O-rutinoside	SARS-CoV-2 protease	(Dubey and Dubey 2020)

The main goal of the present study was to evaluate the antiviral effects of a nano-biotechnological approach resulting from the combination of these emerging therapeutic strategies. To this end, molecular docking analysis was conducted to design a mix of flavonoids to be adsorbed on functionalized TiO_2_ NPs. Their antiviral activity was evaluated against CHoV-229E and SARS-CoV-2, both viruses belonging to the family Coronaviridae, by measuring the survival of virus-infected cells, viral titration, and protein–protein interaction in the presence of different concentrations of functionalized TiO_2_ NPs. Our experimental results indicate that flavonoids adsorbed onto functionalized TiO_2_ NPs (FTNP) exhibit a dramatic concentration-dependent antiviral effect against COVID-19. Based on our spike glycoprotein–mediated cell-to-cell fusion results, a molecular mechanism of action is proposed.

## Material and methods

### Molecular docking studies

SARS-CoV-2 spike glycoprotein (open and closed configurations; PDB (Berman et al. 2000) ID codes 6VYB and 6VXX (Walls et al. 2020), respectively) with the flavonoid ligands, hesperetin 7-rutinoside (H7R) and flavanone-7-O-glucoside (F7G) (PubChem ID: 10,621, 442,428, respectively) were studied using the open-source program Autodock Vina (Trott and Olson 2010). Ligands with lower energy conformations were built with MarvinSketch (20.20 from Chemaxon) (ChemAxon 2019). Structures along with all the analyzed ligands were set up in Chimera UCSF (Pettersen et al. 2004) using the Autodock Vina plugin. Glycosylated conformations were obtained from CHARMM-GUI web server (Lee et al. 2016) and aligned with spike closed state conformation (6VXX) to use the same values on Autodock Vina. Grid box center was placed on coordinates: (220.18, 209.02, 244.48) and box sizes of 124.03 × 132.00 × 70.24 Å^3^ for 6VXX and 124.03 × 132.00 × 81.11 Å^3^ for 6VYB (increment of 10 Å on *Z*-axis was due to its higher length on this axis) were used. Box size was chosen to ensure the entire protein domains of open and close states were included. One hundred independent runs for every compound were conducted for each protein conformation state. Preferred conformation from all the independent runs was obtained by comparing all analytical outputs to the first one and calculating its root mean square (RMS) deviation. An RMS value of 2 Å was used as a cutoff to group all the conformations.

### Molecular dynamics studies

A representative docking snapshot was chosen for molecular dynamics simulation of the closed conformation state (6VXX). To this end, three molecular dynamics simulations were conducted: free protein, protein/H7R, and protein/F7G in the elected conformation from molecular docking. All systems were preprocessed using the input files obtained from CHARM-GUI server and simulated with the free version of the molecular simulation program CHARMM44b2 (Brooks et al. 2009). A cubic solvation box was built around each molecular system with a 10-Å margin from the longest axis. A molecular dynamic of 100 ns was conducted using 2-fs increments at 310.15°K, in a 0.15 M KCl aqueous solution with a pH of 7. All systems were done using GROMACS2019.6 (Abraham et al. 2015) with CHARMM36 parameters (Huang et al. 2017). Cluster analysis was performed using a gmx cluster command with a cutoff of 1.75 Å with the GROMOS method. Interaction maps were created with Maestro (Schrödinger 2021).

### NPs preparation and functionalization

TiO_2_ NPs were prepared by an adsorption process as previously described (León 2014, 2016, 2019). The resulting TiO_2_ particles had a size ranging between 1 and 100 nm. Functionalized TiO_2_ NPs were obtained by sequential addition of solutions containing the functional groups (i.e., hydroxyl, phosphate, sulfate, chloride, amino, methyl, and folate). These solutions were slowly dripped into the NPs suspension that was constantly stirred at 400 RPM. Between each solution, the suspension was allowed to settle for 30 min, to favor the complete adsorption of the functional groups to the NPs. These functionalized NPs were characterized by X-ray diffraction (XRD). XRD measurements show that TiO_2_ NPs exhibit the anatase structure with an average grain size of 2 (± SD) nm. This size was confirmed by transmission electron microscopy (TEM) (Figure S4).

### Extraction of flavonoids and terpenes

Flavonoids and terpenes were extracted in two steps, ethanolic and filtration, as previously described (León 2014, 2016, 2019). Briefly, 70% ethyl alcohol solution was first slowly added to the source of flavonoids and terpenes (seeds, leaves, peels, and shells of selected fruits, like grape, tangerine, orange, grapefruit, lemon, and guava) while it was constantly stirred at about 400 RPM, at a temperature between 30 and 50 °C, for 24 to 48 h. Then, the organic residues recovered during the first stage were mixed with distilled water, placed in a steam distillation apparatus, and heated between 100 and 130 °C. The resulting vapor was transferred to another flask containing the herbal residue and maintained at a temperature between 40 and 60 °C. The resulting liquid was mixed with the one obtained in the first step. The final mixture was incubated at room temperature for 12 h. Extract composition, shown in Table 2, was determined by FT-ICR MS (BRUKER model: solariX) equipped with quadrupole detection (Figure S5).

**Table 2:** Composition of the organic extract and its mass spectrometry data

Flavonoid	Analyte mass	Fragment ions (*m/z*)	Adduct
Hesperetin 7-rutinoside (H7R)	610.6	773.50, 774.51, 775.51	[Glycosil + H]
Flavanone-7-O-glucoside (F7G)	581.17	707.59, 708.59, 709.60	[-H^+^ + ^65^Cu^2+^ + 2CH3OH]
**Terpene**			
Limonene (1-methyl-4-prop-1-en-2-ylcyclohexene)	136.24	137.13	[M + H]
Pinene (2,6,6-trimethylbicyclo[3.1.1]hept-1-ene)	136.24	137.13	[M + H]
Caryophyllene ((1R,4E,9S)-4,11,11-trimethyl-8-methylidenebicyclo [7.2.0]undec-4-ene)	204.36	205.19	[M + H]
Linalool (3,7-dimethylocta-1,6-dien-3-ol)	154.25	155.14	[M + H]
Citronellol (3,7-dimethyloct-6-en-1-ol)	156,27	157.15	[M + H]

Hesperetin 7-rutinoside and flavanone-7-O-glucoside were obtained from Sigma-Aldrich (Cat. 1,304,377 and 91,842 respectively).

### Adsorption process

Organic extracts particles of molecular size ranging between 0.3 to 10 nm were adsorbed to the functionalized TiO_2_ NPs surface through an impregnation process as previously described (León 2014, 2016, 2019). This adsorption procedure requires a molecular surface area of the oxide-containing substrate to be ≥ 50 m^2^g^−1^. For the adsorption, functionalized TiO_2_ NPs suspension was gradually added into a flask containing an aqueous solution of 70% extracts, under continuous stirring (100 ~ 400 RPM) for 24 h. The flavonoids adsorbed TiO_2_ NPs (FTNPs) were stored at room temperature until used.

### Viruses and cells

#### Cell culture

Cells were cultured in 25-cm^2^ petri dishes. Vials containing 1 mL of cells suspension (Table 3) were thawed, followed by the addition of 4 mL Gibco Minimum Essential Media (MEM) 1 × and 10% fetal bovine serum. Then, cells were incubated under standard culture conditions (37 °C in a humidified atmosphere containing 5% CO_2_). Cells were detached immediately after reaching 100% confluence with 10% of trypsin, split in two and 10% bovine fetal serum was added to stop trypsinization. MEM 1 × (4 mL) was added and cells were incubated under standard culture conditions.

**Table 3:** Viral strains and cell lines used in this study

Collection	Virus strain	Classification
ATCC (VR-740)	CHoV-229E	Coronaviridae, coronavirus
Virus isolated in the Navy Hospital, Mexico GISAID Accession ID: EPI_ISL_8930099	SARS-CoV-2 Delta variant (B.1.617.2)	Coronaviridae, betacoronavirus, severe acute respiratory syndrome-related coronavirus 2
**Collection**	**Cell line**	**Derivation**
ATCC (CCL-7)	LLC-MK2 Original	(*Macaca mulatta*) rhesus monkey
ATCC (CCL-81)	Vero E6	(*Cercopithecus aethiops*) African green monkey kidney monkey
ATCC (CCL-171)	MRC-5	(*Homo sapiens*) lung tissue of a 14-week-old male fetus
ATCC (CCL-61)	CHO-K1	(*Cricetulus griseus*) hamster

#### Viral culture

Cells were grown in 75-cm^2^ boxes until reaching 80% confluence. They were infected with the corresponding virus (Table 3) and incubated under standard culture conditions. Cells showing signs of cytotoxicity (i.e., syncytia formation) were scraped for cell lysis and release of viral particles. Cells lysate and viral particles were collected and stored at − 72 °C until being used.

### Solutions

Minimum essential medium (MEM 10 ×) with Earle’s salts, L-glutamine, and non-essential amino acids without NaHCO_3_. Fetal bovine serum, free of endotoxins, mycoplasmas, and viruses, 0.1 mL, sterilized by filtration. Trypsin 0.25 M and versene 0.1% solutions were made in phosphate saline solution without added Ca^2+^ and Mg^2+^.

### Lytic plaque viral titration

Cells grown in 24-well plates until reaching 80% confluence were washed with isotonic saline and added with 1 mL MEM. Immediately before the assay, the medium was replaced with virus-containing fresh MEM at different dilutions. For each of the four plates rows, viral harvest was added to each well at progressively higher dilutions (in a 1:9 dilution for the first well), up to the last one which contained no virus (negative control). After 2-h incubation under standard culture conditions, cells were washed with saline solution, added with 1 mL MEM, and incubated for 48 h under similar conditions. After this period, cells were washed and 200 µl of alcohol-acetone mixture (1:2) was added for 10 min, the excess was removed and left to dry. Lastly, cells were incubated for 1 min in 1% violet crystal solution, washed with tap water, and the lytic plaques (or presence of syncytia) were examined and counted.

### qRT-PCR

Viral RNA was extracted from culture using ScienCell’s SARS-CoV-2 Coronavirus Real-time RT-PCR (qRT-PCR) Detection Kit (CVPD) and stored at − 80 °C until further analysis. The extracted viral RNA was quantified using the same ScienCell™ detection kit (Cat-7038) and CFX96 and CFX384 Real-Time System (Bio-rad) with a TaqPath™ 1-step qRT-PCR Master Mix, CG, 4 × (Thermo Fisher, Cat-A15299). Three primer/probe sets were used (Cat-7038-N1, 7038-N2, and 7038-N3) that target the conservative regions of coronavirus nucleocapsid (N) gene, the Human RPP30 gene primer/probe set (Cat-7038-RP) that targets exon 1 of human RPP30 gene and serves as a control to assess specimen quality, a non-infectious DNA positive control (Cat-7038-Pos) to ensure reagents and instruments are working properly, and a negative human specimen extraction control (human RNA extract from non-infected samples, Cat-7038-Hsc) for assessing reverse transcription.

### Cell viability

XTT Cell Viability Assay kit (Biological Industries Cromwell, CT, USA) was used per manufacturer’s instructions. Briefly, Vero E6 cells, seeded in 96-wells plates at a confluence of 100%, were infected with the SARS-CoV-2 virus (multiplicity of infection = 1.0) previously incubated with functionalized NPs for 5 min at room temperature. NPs suspension was made at subsequently larger dilutions (1 × 10^−1^ to 1 × 10^−11^). Plates wells were inoculated with NPs by quadruplicate and incubated for 48 h under standard culture conditions. Cellular metabolic activity was measured as optical absorbance at 450 nm wavelength.

### Luciferase-based reporter assay

A standard virus-free, cell-to-cell fusion assay was performed as described previously (Tiwari et al. 2004). Briefly, the target CHO-K1 cells were co-transfected with human ACE-2 receptor (2.0 µg) and the luciferase gene (0.5 µg) using lipofectamine 2000 (Invitrogen, Waltham, MA). In parallel, the effector CHO-K1 cells were transfected with SARS-CoV-2 spike (2.0 µg) and T7 RNA polymerase (0.5 µg). After 24 h post-transfection, effector cells were treated with TiO_2_ NPs and or mock-treated with (1 × PBS) before mixing with the target cells. The extent of cell-to-cell fusion was quantified using a reporter lysis assay (Promega, Madison, WI) 24 h post-mixing of the target and effector cells.

### Syncytia formation

A syncytia formation assay was carried out to determine the effect of TiO_2_ on multinucleated cell formation as previously described (O’Donnell and Shukla 2009). Target cells expressing human ACE-2 receptor (2.0 µg) and 0.5 µg of a plasmid expressing cyan fluorescent protein (CFP) fused with a nuclear localization signal (NLS) (Clontech, Mountain View, CA) were co-cultured with the TiO_2_ pre-treated effector cell expressing spike (2.0 µg) and 0.5 µg of a red fluorescent protein (RFP) fused with a nuclear export signal (NES) (Hu et al. 2005). Syncytia number and images were captured 24-h post-mixing with a 63 × objective on a line-scan confocal microscope (Lecia DMIRE2) equipped with a camera (Lecia TCSSP2).

### Statistics

All tests were done at least in triplicate. GraphPad-Prism® software (version 9.0.0) was used for graphing and statistical analyses. Error bars obtained from quadruplicate testing.

## Results

Based on an extensive literature search for natural compounds with antiviral activity (Boukhatem 2020; Kaul et al. 2021; Orhan and Deniz 2020; Tsuchiya et al. 1985), we discarded those reported to have high cytotoxic effects. Of the remaining compounds, we focused on those with activity on viruses similar to SARS-CoV-2, specifically viruses of the Coronaviridae family. The resulting group consisting of flavonoids and terpenes was then used for molecular docking analyses. The two final ligands, H7R and F7G, were selected since they exhibited the lowest affinity energy, and therefore the highest affinity. In addition, their binding region is located on sites that could be key to the function of the spike protein.

### Molecular docking studies

To investigate the molecular mechanism by which our ligands of interest (i.e., flavonoids or terpenes) interfere with the coupling between the SARS-CoV-2 spike and the human ACE-2 receptor, protein–ligand molecular docking analyses were conducted. Specifically, the affinity energy of the flavonoids or terpenes binding to different possible sites of the spike was measured. Open-and-close configurations of SARS-CoV-2 spike were used as targets for the analyses. A total of 100 independent docking evaluations were conducted with each ligand binding to the spike in both, the open and close configuration, to determine the most favorable position for each compound. All our analyses were based on the most populated conformation for each complex. Table 4 shows the molecular fraction (expressed as percentage) of selected compounds bound to either the open (PDBID: 6VYB) or close (PDBID: 6VXX) configuration of SARS-CoV-2 spike, as well as their corresponding affinity energy.

**Table 4:** Affinity energy (kcal mol^−1^) and percentages (on parenthesis) for the compounds selected in this study

Flavonoid	6VYB	6VXX
Hesperetin 7-rutinoside (H7R)	(35%) − 9.26 ± 0.29	(58%) − 9.89 ± 0.15
Flavanone-7-O-glucoside (F7G)	(33%) − 9.04 ± 0.08	(30%) − 9.40 ± 0.11

**Fig. 1:**
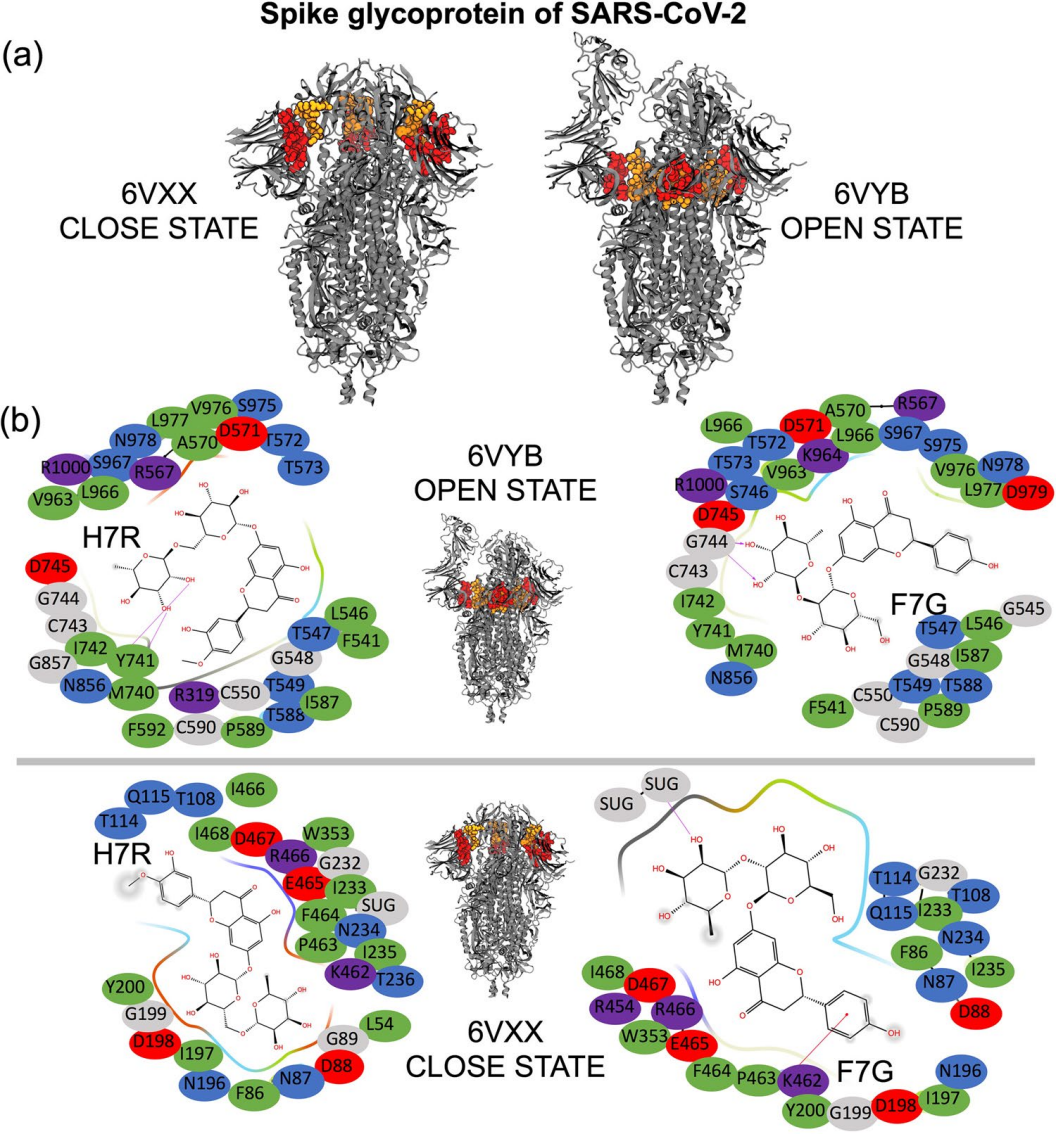
**a** The binding sites of the selected flavonoids (H7R and F7G) to SARS-CoV-2 spike glycoprotein. Distinct conformational of spike glycoprotein chains in RBD closed (receptor inaccessible) and open (receptor accessible) states are shown on orange and red, respectively. **b** H7R and F7G interaction maps with SARS-CoV-2 spike glycoprotein in the open (top panel) and close (bottom panel) configurations. Spike glycoprotein amino acid colors in the interaction maps, represent their properties: negatively charged in red, positively charged in dark blue, hydrophobic in green, and polar in light blue. Molecular docking (MD) studies were conducted using Autodock Vina, MarvinSketch to convert 2D to 3D structures, visual molecular dynamics (VMD) was used to generate molecular images; while computational modeling using Maestro (Schrödinger 2021–4) were used for the detection of molecular ligand-receptor interactions

As indicated by the low-affinity energy values, H7R and F7G exhibited high binding affinity. All of the spike protein residues located within 5 Å from both ligands were included in the analysis. These residues were also mapped in the spike structure (Fig. 1a), as well as in their corresponding interaction maps (Fig. 1b).

The molecular structures shown as ribbon-spheres diagrams were generated with VMD v.1.9.4a43 (Humphrey et al. 1996). This molecular modeling software allowed us to choose or hide specific regions of the molecules to emphasize their binding properties. However, the energy calculations were conducted with the entire molecule. We hypothesized that these residues in the open structure are critical in the dynamic behavior of the spike protein.

These critical spike amino acids are shown in Fig. 1b, interacting with the flavonoid ligands F7G and H7R in the open-and-closed configurations of the protein. The specific regions of the protein are indicated on red and orange spheres.

**Fig. 2:**
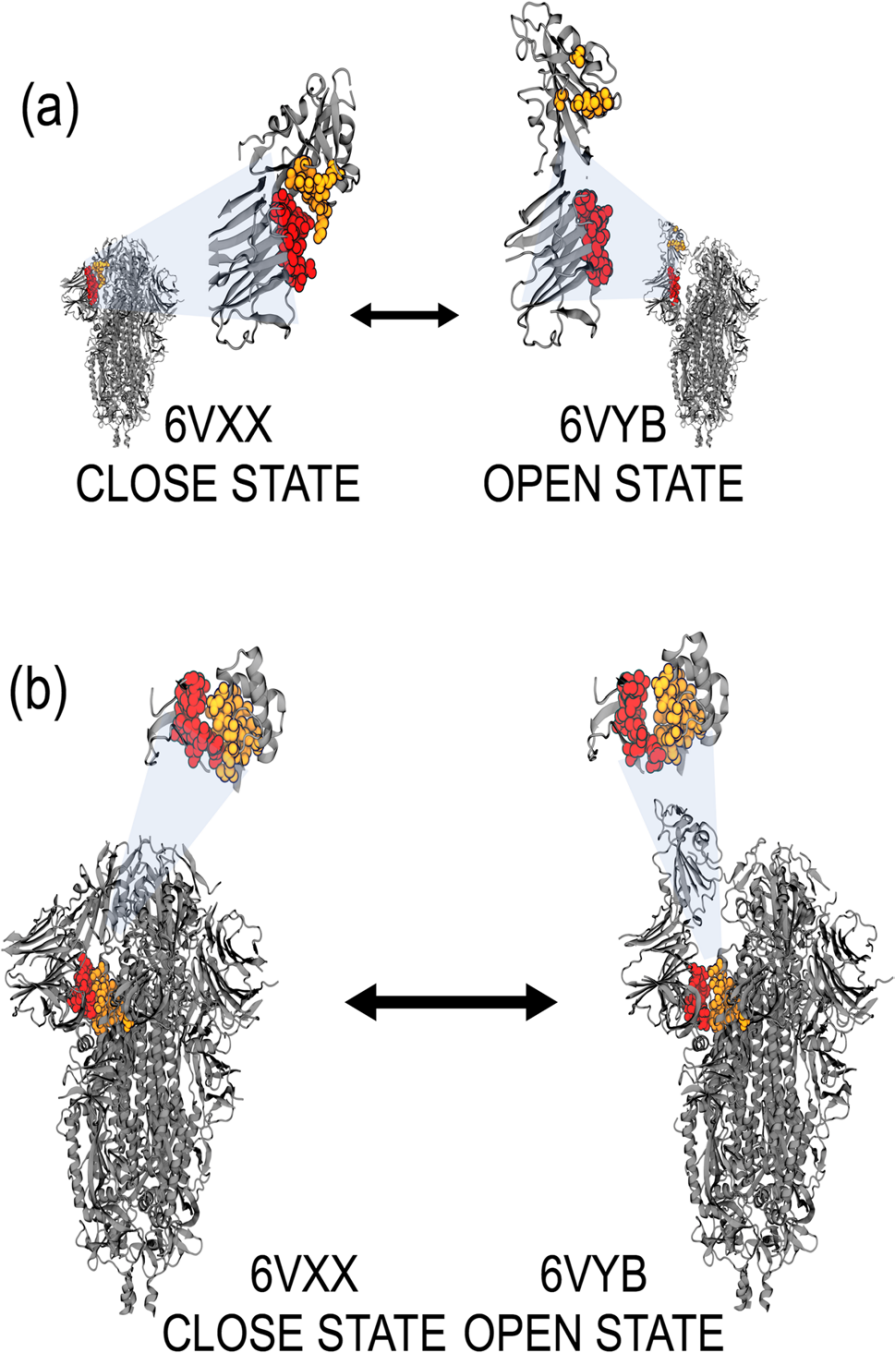
**a** Residues mapped on chains B and C for the open and close configurations of the SARS-CoV-2 spike glycoprotein. Green spheres show the regions where H7R and F7G interact with the bottom of the S1 domain, while the orange spheres show the regions in the top of the S1 domain (near the ACE-2-binding site). Notice that in the open state, the orange spheres appear separated indicating that a compound bound to these regions may disrupt its movement avoiding the correct position to interact with ACE-2. **b** Chain B (white) of 7A93 structure (SARS-CoV-2 S with 2 RBDs erect) was superimposed with chain B (blue) of 6VYB. Residues F318, P295, W633, R634, and Y636 are illustrated with green spheres. H7R (blue) and F7G (red) spheres binding sites are also shown in chain B

Previous studies indicate that the ACE-2 receptor-binding domain (RBD) of the SARS-CoV-2 spike is formed by one half of each of the S trimers (Benton et al 2020). This RBD is shown in Fig. 2a, where the lower part of S1 is located in the binding site of H7R and F7G of the open state configuration (6VYB; residues 541, 545–550, 567, 570–573, and 587–590 of one chain and residues 740–746, 856, 857, 963, 966, 967, 975–977, 979, and 1000 of the opposite chain). Our molecular docking analysis revealed two specific binding regions for H7R and F7G in the open and close states, which are formed by the joint between the bottom of the S1 domain and by the top of the S1 domain. Specifically, these regions are formed by residues 54, 86–89, 108, 114, 115, 196–200, 232–236 on any chain and residues 353, 454, 462, 463, 464, 465, 466, 467, 468 of the adjacent chain.

These results would suggest that both flavonoids may disrupt the interactions between SARS-CoV-2 spike and ACE-2, through binding to residues located in different chains. Specifically, F7G may interfere with the interactions between ALA570:B and VAL963:C, ASP571:B and SER967:C, THR572:B and ASN856:C and VAL963:C on the open state and between PRO463:B and ASP198:C, PHE464:B and ASP198:C, ARG466:B and GLN115:C and GLY232:C, ILE468:B and GLN115:C in the close state.

Based on our results, we expected that H7R and F7G bind to the top of the spike protein near the binding region with ACE-2. Even when they do not seem to block the spike protein-ACE-2 interaction, they may interfere or inhibit the necessary movement of the top region of the spike protein (Fig. 2b) by interfering with the residues mentioned before. The inhibition of this movement may block the correct exposition of the binding region with ACE-2, thereby preventing coronavirus-19 infection. Furthermore, H7R and F7G bind to the spike protein in the open-state near the N-terminal domain, which is located near the region previously reported by others (Benton et al. 2020). These results indicate that both ligands may disrupt the movement needed for spike protein S1 domain to separate from S2 core Fig. 2b. Together, both effects (at the top and bottom on S1) may inhibit COVID-19 infection in human cells by interfering with the native movement of the spike protein.

### Molecular dynamic

Molecular dynamics were conducted to test the stability of the protein–ligand complexes found with our molecular docking analysis (see supplementary material). Simulations were performed in the following three conditions: free protein, protein/H7R, and protein/F7G. All systems were simulated during 100 ns. To evaluate the complex stability, α-carbon root mean square deviation (RMSD) from the initial structure was calculated. RMSD evolution during the simulation is shown in Figure S1, where it can be observed regions with a drifting behavior in the final 10 ns (6VXX). A root mean square fluctuation (RMSF) analysis of the final 40 ns of each simulation showed that there are regions with more movement than others (data not shown). The more mobile regions correspond to the first and last 15 amino acids (amino and carboxyl-terminal) and to a loop (676–689) in the middle section of the SARS-CoV-2 spike structure. Figure S1 shows the RMSD calculation without these more mobile regions (reduced α-C), which supported the conclusion that the RMSD behavior observed on all α-carbons was due to the movement of these regions. Based on this observation, the final 40 ns of each simulation was used for the subsequent analysis.

Cluster analysis was conducted to find a set of representative structures for each simulation. The selected cluster had at least 68% of the analyzed conformations (free protein 68%, protein/H7R 92%, and protein/F7G 68%). The middle structure of each cluster was analyzed for any protein–ligand interactions. Figure S2 and figure S3 show the interaction maps and three-dimensional interactions between 6VXX and the ligands H7R and F7G. We concluded that those interacting amino acids found with docking analysis that remained in close molecular interaction throughout the molecular dynamics simulation, constitute (at least partially) the binding site for H7R and F7G. Specifically, those amino acids were, for H7R: F464, E465, R466, D467, and I468 in chain A and T114, Q115, G232, I233, N234 on chain B; while for F7G: R466 in chain B and Q115, G232, and I233 in chain C.

Some interactions from molecular docking were lost in the molecular dynamic simulations, as expected from the molecular movement of sugar chains, interacting ions, and the protein itself. However, it is important to note other interactions with both ligands exhibited a high level of stability in the binding site. The additional interactions found in the simulations and shared by both ligands were V130, T167, F168, P230, I231 on one chain and N354, R355, K356, R357, and Y396 on the subsequent chain. H7R formed hydrogen bonds with I231, R355, R466 (as a donor of hydrogen bond), and with R466 and T167 (as an acceptor of hydrogen bond) (Fig. S2). Meanwhile, F7G interacted with Q115, E169, P230, I231, and R355 (as a donor of hydrogen bond) and with T167 and R466 (as an acceptor of hydrogen bond). As noted, the number of hydrogen bonds was higher in H7R than in F7G. Hydrogen bond analysis along the 40 ns of molecular simulations showed that H7R had 5.78 ± 1.29 and F7G had 2.89 ± 1.40 hydrogen bonds. These results showed that both ligands, H7R and F7G, bind stably to SARS-CoV-2 spike.

### Effects on CHoV-229E and SARS-CoV-2 infectivity

We investigated the FTNPs effects on CHoV-229E infectivity under in vitro conditions. To this end, confluent MRC-5 cells were infected with CHoV-229E at an infectious multiplicity of 1.0 (M.O.I. = 1.0) and incubated for 72 h. In parallel experiments, untreated MRC-5 cells (seeded at a density of 1.2 × 10^6^/dish and cultured for the same time) were used as a negative control. Under these conditions, CHoV-229E-infected cells exhibited clear signs of infection, which consisted of cellular rounding, refractiveness, detachment, and loss of confluence. Negative control (uninfected) cells did not show any of these signs. In a similar assay using VERO.E6 cells, it was observed that the virus decreased six orders of magnitude when pre-incubated with FTNP (from 6 × 10^10^ to 3 × 10^4^ PFU). The assays were performed in triplicate using 24-well plates and minimum essential medium (MEM) supplemented with 1.5% carboxymethyl cellulose (Figure S6).

To determine if FTNPs could prevent or reduce CHoV-229E replication, 1.2 × 10^6^ viral particles were incubated first with FTNP for 60 s at a 1:4 dilution and added to 24 h cultured MRC-5 cells. After 48 h, MRC-5 cells still exhibited signs of infection (i.e., cellular rounding, detachment, loss of confluence; better known as cytopathic effect) but to a considerably lesser extent. These signs of cytopathic infection, however, essentially disappeared when CHoV-229E were pre-incubated with FTNP at higher concentration (i.e., 1:2 dilution). To verify the level of viral infection under these different conditions, the presence of CHoV-229E was determined by qRT-PCR assays. The presence of CHoV-229E in infected cells in the absence of FTNP was confirmed, while in the presence of FTNP qRT-PCR assay revealed no viral residues.

**Fig. 3:**
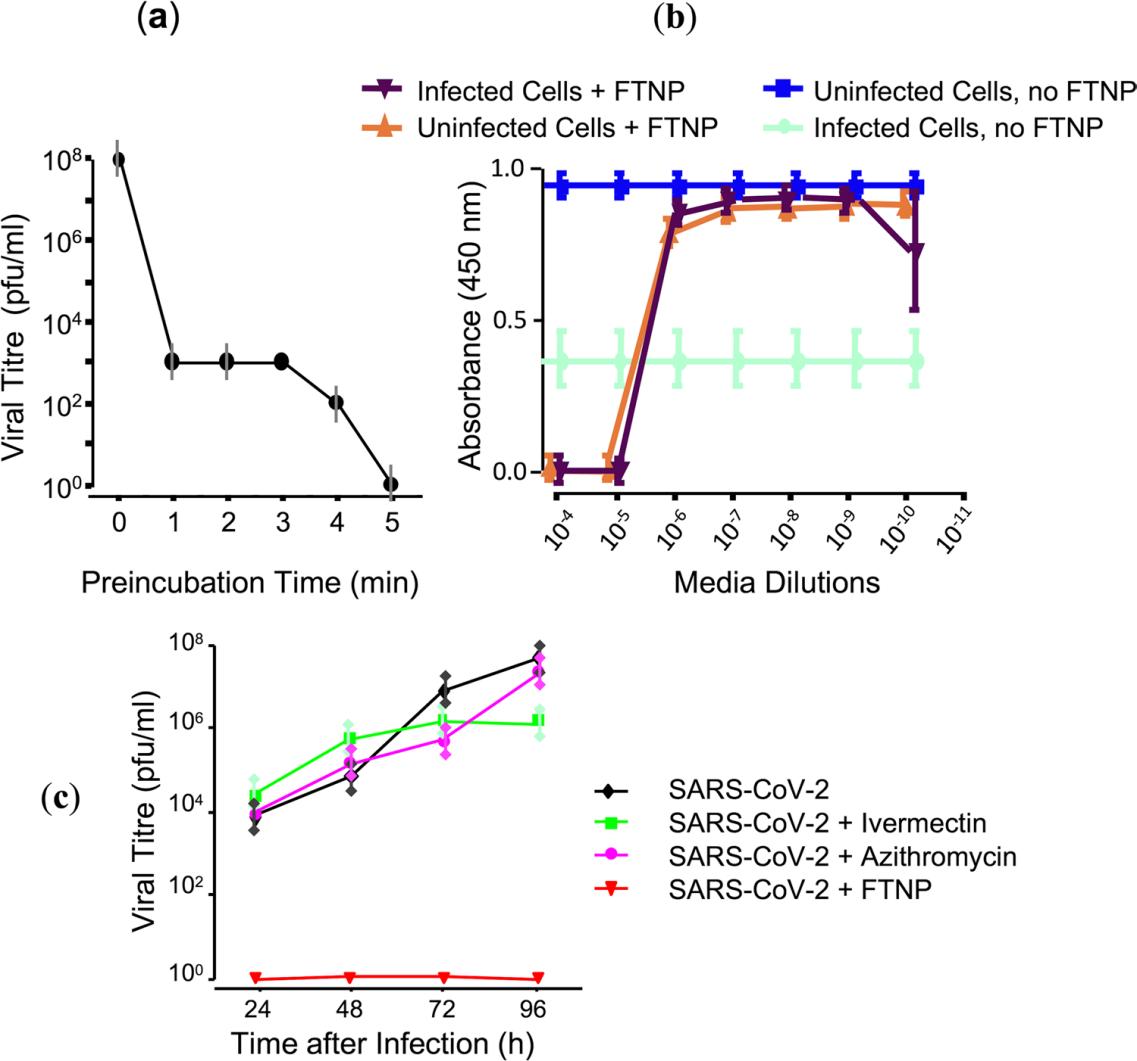
The effect of functionalized nanoparticles (FTNP) on SARS-CoV-2 replication estimated by XTT assays in VeroE6 cells (measured as plaque-forming units; pfu). **a** SARS-CoV-2 viral titer as a function of the pre-incubation time with FTNP is shown. **b** Cell viability infected with SARS-CoV-2 in the presence and absence of FTNP. The dilutions on the *X*-axis represent the order of magnitude of viral load. **c** A study comparing the effect of FTNP using different other antimicrobial agents (ivermectin and azithromycin) on SARS-CoV-2 replication. In this experiment, the SARS-CoV-2 was pre-incubated with FTNP (at a dilution of 2 × 10^−7^) or ivermectin (5 µM), or azithromycin (5 µM), while VeroE6 cells infected with SARS-CoV-2 in absence of any drug was used as a positive control

The effectiveness of FTNPs to reduce/prevent cellular infection by SARS-CoV-2 was also evaluated by measuring cell viability. To this end, VERO.E6 cells were chosen for being among the most permissive cells for in vitro SARS-CoV-2 viral replication. FTNP was pre-incubated in MEM 1X medium for 0 to 5 min with SARS-CoV-2 and the viral titer was quantified at each pre-incubation time (Fig. 3a). These results showed a clear reduction of SARS-CoV-2 viral replication as the FTNP pre-incubation time was increased to a point where 5 min pre-incubation essentially prevented any viral growth. To evaluate the FTNP antiviral effects on cellular survival, the cellular metabolic activity was measured at different FTNP concentrations by optical absorbance (Fig. 3b). In the absence of FTNP, SARS-CoV-2-infected cells showed low metabolic activity levels as absorbance values below 0.5 indicate (Fig. 3b). These absorbance values were interpreted as low cellular viability resulting from the viral infection. On the other hand, absorbance values close to 1 observed in uninfected cells in the absence of FTNP were interpreted as high cellular viability (Fig. 3b). However, when SARS-CoV-2-infected cells were incubated with FTNP at dilutions larger than 1 × 10^−6^ for 5 min, absorbance values close to 1 were detected, indicating high metabolic activity (Fig. 3b). Interestingly, the protective effect of FTNP against SARS-CoV-2 infection started to decline at dilutions larger than 10^−9^, as judged by the reduction of the absorbance value to about 0.75. It is important to notice, however, that SARS-CoV-2-infected cells incubated with FTNP at dilutions lower than 1 × 10^−6^ showed essentially no metabolic activity (absorbance values of 0.0). This was an essentially identical pattern to that shown by uninfected cells treated with FTNP (Fig. 3b). These observations indicated that at low concentrations (≥ 10^−6^ dilution), FTNP effectively prevents viral infection to levels essentially like those observed in uninfected cells. However, high concentrations of FTNP (≤ 10^−5^ dilution) induce cytotoxic effects. This was also observed with individual flavonoids (Figure S7).

To further define the protective effects of FTNP against SARS-CoV-2 infection, infectious viral material was quantified in infected cells at different times of culture. To this end, lytic plaque assay was conducted in infected cells incubated with FTNP and compared to those without it. The results of these experiments indicated that SARS-CoV-2-infected cells pre-incubated with FTNP showed viral titration values close to 0, even after 96 h of culture (Fig. 3c). In contrast, in cells that were cultured without FTNP, viral titration values increased exponentially as a function of culture time (Fig. 3c). These results supported the notion that FTNP effectively prevents cellular infection by SARS-CoV. As a comparison, other agents such as ivermectin and azithromycin with reported antiviral activity against COVID-19 (Yang et al. 2020; Echeverría-Esnal et al. 2021) were also evaluated with lytic plaque assay (Fig. 3c; ivermectin; azithromycin). The results of these assays indicate that although ivermectin did not reduce viral titer values, it was able to prevent viral increase after 72 h. In contrast, in the presence of azithromycin viral titer values continued to increase, reaching similar values to those in the absence of any antiviral agents at 96 h.

On the other hand, in vitro activity against SARS-CoV-2 was measured with the individual flavonoid and terpene extract compounds present in FTNP and the only ones that showed activity were the two flavonoids; H7R and F7G (results shown in Figure S4).

### SARS-CoV-2 spike glycoprotein–mediated cell-to-cell fusion

**Fig. 4:**
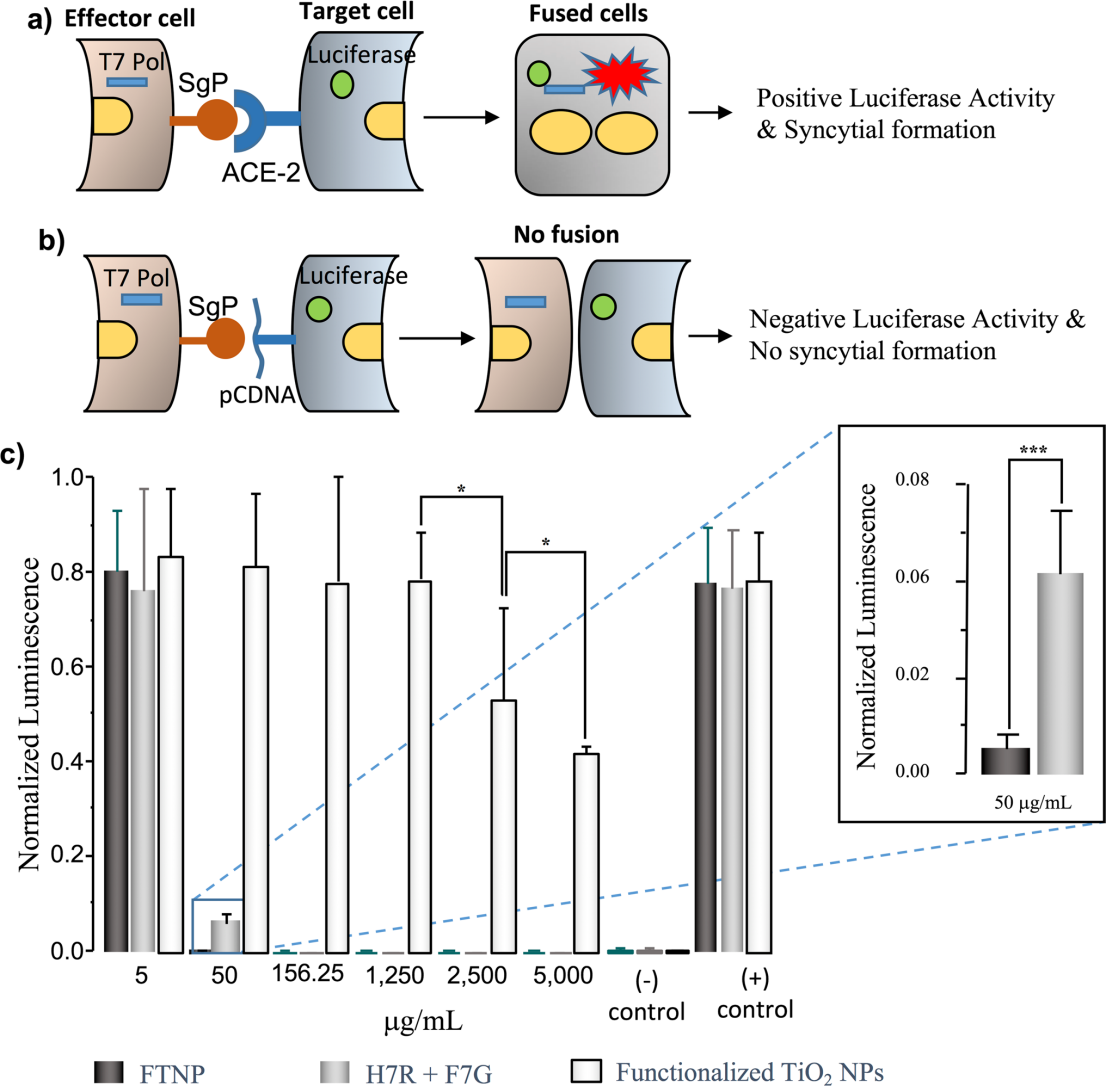
**a–b** Schematic representation of the reporter luciferase-based SARS-CoV-2 spike glycoprotein-mediated cell-to-cell fusion occurs in presence of ACE-2 receptor (panel **a**) but not in the absence of ACE-2 (panel **b**). An effector cells co-expressing SARS-CoV-2 spike glycoprotein together with T7 polymerase co-cultured with the target cell co-expressing human ACE-2 receptor with luciferase gene (panel **a**) and or an empty vector pCDNA3.1 plasmid with luciferase plasmid (panel **b**). Luciferase activity was measured 24 h co-culture of the effector and target cell in presence and absence the nanoparticles. Relative luciferase activity was determined by using a luminometer. **c** Normalized bioluminescence of spike glycprotein-ACE-2 receptor interaction during cell-to-cell fusion in the presence of TiO_2_ NPs (), H7R + F7G (), and FTNP (functionalized TiO_2_ NPs + H7R + F7G) () were quantified. Bars represent mean ± SD, *n* = 9 (*p < 0.05; ***p < 0.0005; two-tailed unpaired *t* test)

To confirm the mechanism(s) of the FTNP protective effects against SARS-CoV-2 infection, a luciferase assay was conducted in co-transfected CHO-K1 cells (as described in the Methods section). The underlying premise tested here was that SARS-CoV-2 infection is triggered by the specific protein–protein interaction between SARS-CoV-2 spike and ACE-2 receptor in the host cells. CHO-K1 cells were co-transfected with luciferase or T7 RNA polymerase and either the SARS-CoV-2 spike or the ACE-2 receptor (Fig. 4a, b). Under these conditions, intercellular fusion was quantified as bioluminescence in the presence of either functionalized TiO_2_ NPs, the mix of flavonoids H7R + F7G, or the combination of both, functionalized TiO_2_ NPs, H7R, and F7G (FTNP). The results of these assays, shown in Fig. 4c (mean ± SD, *n* = 9), indicate that all compounds induced inhibition of cell fusion, suggesting an important and specific protective effect against SARS-CoV-2. As the compound concentration increased, the amount of luminescence decreased as well. The biggest effect was observed when cell fusion was evaluated in the presence of either the flavonoids alone (H7R + F7G) or the flavonoids plus functionalized TiO_2_ NPs (FTNP). Notice that at 50 µg/mL, the extent of bioluminescence was essentially eliminated by the two agents, but the reduction was about sevenfold more pronounced with FTNP than with H7R + F7G (*p* < 0.0005). However, when the assay was conducted in the presence of functionalized TiO_2_ NPs alone (FTNP without flavonoids), cellular fusion was partially reduced and only became significant at higher concentration (i.e., 3 mg/mL). On the other hand, the non-functionalized TiO_2_ NPs exhibited no inhibitory effects on cell fusion at any of the tested concentration (data not shown). As a reference, control experiments were conducted in parallel. Cell fusion was evaluated under similar experimental conditions but in the absence of any kind of NPs: functionalized TiO_2_ NPs, H7R + F7G, or FTNP (positive control).

Therefore, cell fusion was not impaired, and bioluminescence showed its highest values. As a negative control, the experiments were carried out with non-transfected CHO-K1 cells and in the presence of functionalized TiO_2_ NPs and H7R + F7G. Under these conditions, cell fusion could not occur due to the lack of specific protein–protein interaction; therefore, no bioluminescence was generated regardless of the presence of inhibitors.

**Fig. 5:**
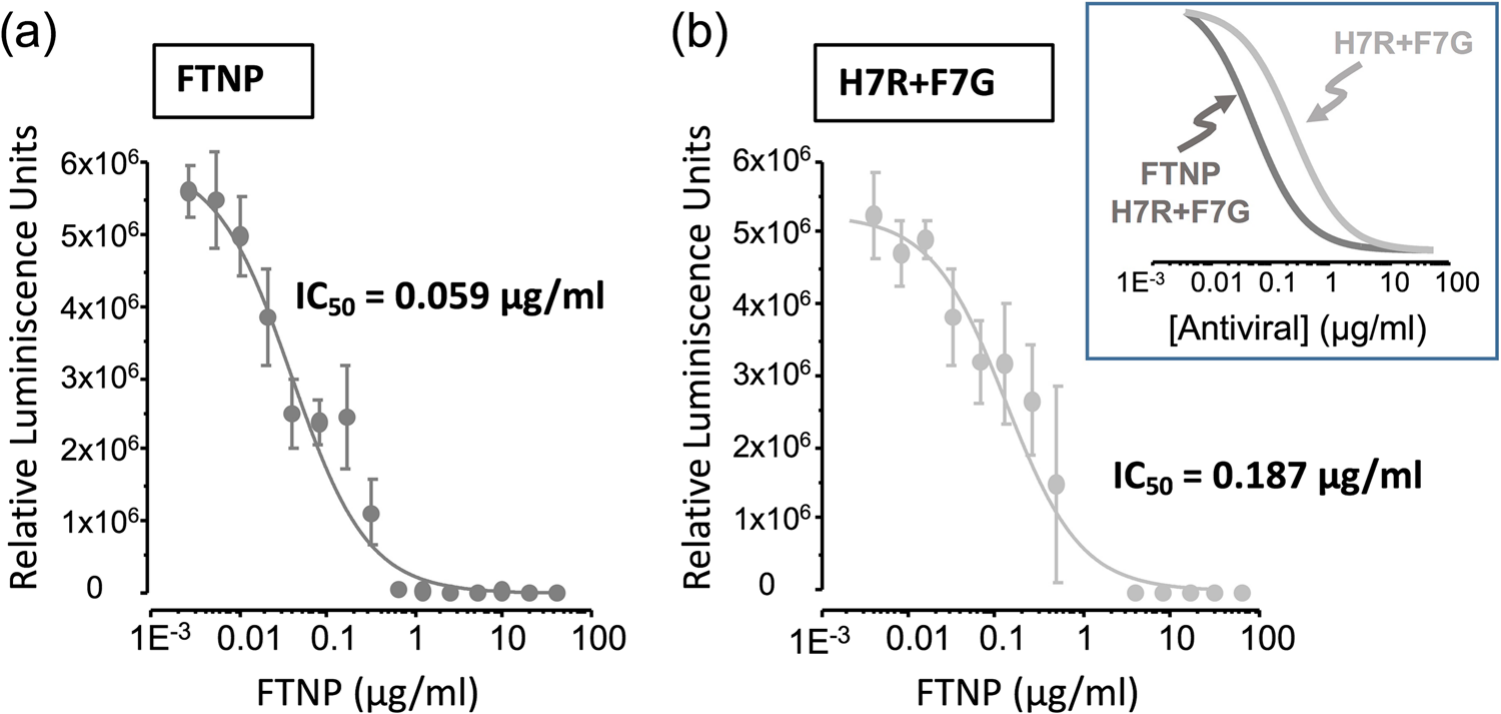
The antiviral effect of FTNP in comparison to flavonoids. Bioluminescence dose–response curve is generated in the presence of FTNP (**a**) or H7R + F7G (**b**). Data points were fitted with a Hill equation of the form: *f*(*x*) = 1 / [1 + (IC_50_/*x*) *n*], where *f*(*x*) is expressed as RLU, *x* represents [antiviral], IC_50_ is the [antiviral] required to yield 50% of the maximal RLU, while *n* is the Hill coefficient. Best-fitting curves were obtained with an IC_50_ of 0.059 µg mL^−1^ for FTNP + H7R + F7G, and 0.187 µg mL^−1^ for H7R + F7G (*p* < 0.005; two-tailed, unpaired *t* test), and with an *n* of − 0.73 and − 0.96, respectively. Bars represent mean ± SD, *n* = 9. Inset in panel **b** shows normalized overlapped fitting curves for these conditions

To determine the effective concentration range, dose–response relationships were defined separately for the whole mixture (FTNP) and the flavonoids mix (H7R + F7G). The results of these experiments shown in Fig. 5 revealed that both combinations were well described by a sigmoidal function with a Hill coefficient of − 0.73, but in the presence of FTNP, there was an increase in the apparent affinity, as indicated by a threefold decreased in the IC_50_ (from 0.187 µg mL^−1^ with H7R + F7G to 0.059 µg mL^−1^ with FTNP; *n* = 9; *p* < 0.005). Taken together, these results supported the notion that the antiviral effects of the FTNP most likely take place by interfering with the interaction between SARS-CoV-2 spike and the ACE-2 receptor.

Compared to other nanoparticle compounds, our FTNPs have the advantage of being significantly less toxic, since the large majority of other are made of heavy metals (e.g., copper, zinc, silver) (Mishra et al. 2011, 2019). In fact, TiO_2_ is an approved ingredient for human consumption as a food additive (Tiwari et al. 2004). Another important advantage is that the FTNP functionalization minimizes their cytotoxicity, thereby eliminating the need for photoactivation required to enhance their antiviral potential.

Even though FTNPs antiviral efficacy on SARS-CoV-2 needs to be determined in humans for potential use as a therapy, this knowledge will be critical for drug development by correlating effective binding for specific antivirals. Although sometimes it is more effective to repurpose existing broad-spectrum drugs to fight emergent new diseases like COVID-19, there are numerous advantages when designing a new specific antiviral drug, such as efficacy and specificity. Our work demonstrates the enormous potential of nanotechnology for therapeutic use against COVID-19.

## Discussion

Designer intercepting multiligand interactions during viral entry provides an excellent platform to mitigate the current SARS-CoV-2 pandemic (Sanna et al. 2022; León-Gutiérrez et al. 2021). In the current study using molecular docking strategy, we first designed FTNP and further characterized for their antiviral potential against SARS-CoV-2. The experimental results generated from the virus infectivity and spike mediated cell-to-cell fusion assays showed that FTNP indeed exhibits a powerful antiviral effect against SARS-CoV-2 (Fig. 3, 4). Furthermore, the affinity energy calculations together with the molecular docking analysis indicated that the flavonoids H7R and F7G bind to specific regions of the SARS-CoV-2 spike located outside of the receptor-binding domain, both in the open and close configurations of the protein (Figs. 1, 2). In addition, our molecular docking analysis further indicates that the flavonoids binding to the close state configuration may interfere with the erection mechanism involved in the transition to the open state, which is a critical requirement for binding to the ACE-2 receptor (Teruel et al. 2021). Interestingly, we further noted that the flavonoids binding to SARS-CoV-2 spike are not only very specific but highly stable, as evident from our molecular dynamic studies. The flavonoid-binding sites were found to be located outside the RBD of the spike, which seems extremely relevant as the antiviral effects of FTNP are likely to be preserved in the event of genetic variation in the spike (e.g., omicron and omicron B.2) as currently observed worldwide (Hoffmann et al. 2022).

Earlier computational modeling studies at the molecular level have predicted that several phytochemicals including hesperidin either dock on ACE-2 receptor and or on the complex of SARS-CoV-2 spike protein and human ACE2 (Basu et al. 2020); however, in our in vitro–based studies, we found a contrasting effect of flavonoids during SARS-CoV-2 entry. As evidenced by our studies, the pre-treatment of FTNP with the virus but not with the target cells showed the inhibition of viral entry (Fig. 3a). Taken together, our results highlight that FTNP interacts with the SARS-CoV-2 spike protein but not with the ACE2 receptor.

Different types of antivirals vary mechanistically and interfere with different stages of the viral life cycle (Kausar et al. 2021). Thus, during the infective process, the infected cell releases new viral particles which initiate a new infective process. It is at this time that these new viral particles could encounter the FTNP and prevent them from continuing to infect new target cells. Our results indicate that the antiviral effect of FTNP is at the level of virus entry into the target cell (Fig. 3a).

Our observation that FTNP has antiviral effects on HCoV-229E, and SARS-CoV-2 is significant because there are seven other coronavirus (CoV) strains that are pathogenic to humans, from which four (HCoV 229E, HCoV NL63, HCoV HKU1, and HCoV OC43) can cause flu-like symptoms (V’kovski et al. 2021). Since all CoV share similar structural arrangements (namely, a single-strand RNA genome and a virion containing four main structural proteins: nucleocapsid, transmembrane, envelope, and spike) (Naqvi et al. 2020), it is reasonable to assume that the FTNP antiviral effects result from similar mechanism(s) of action.

**Fig. 6:**
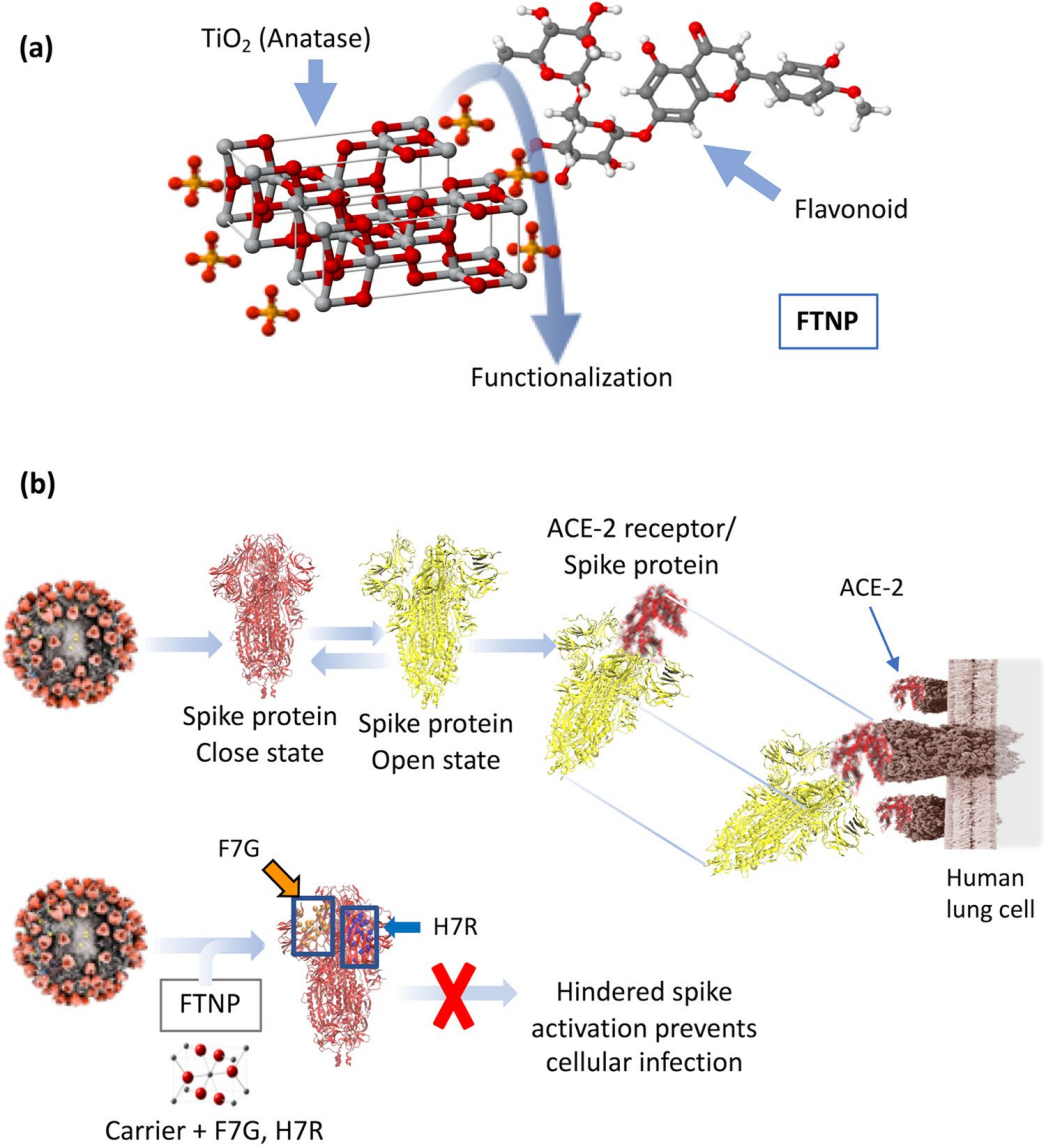
**a** Schematic conformation of FTNP. **b** Proposed model of the FTNP mechanism of action

Since spike and ACE-2 are a known ligand and a receptor pair during SARS-CoV-2 cell entry (Wang et al. 2020), we took advantage by using reporter luciferase-based SARS-CoV-2 spike mediated cell-to-cell fusion assay. FTNP showed an impressive dosage-dependent effect in blocking spike mediated cell-to-cell fusion which suggests that it likely interferes with the critical interactions between the SARS-CoV-2 spike with the ACE-2 receptor during host cell interactions (Fig. 4C). Furthermore, the potent antiviral activity of FTNP observed when pre-incubated with the spike expressing cells (Fig. 3a) indicates that the SARS-CoV-2 spike protein-ACE-2 interaction is disrupted in presence of FTNP. In summary, our study shows that the functionalized TiO_2_ NPs coated with the flavonoid’s carrier, bind to SARS-CoV-2 spike which in turn affects spike recognition to the ACE-2 receptor, thereby preventing the virus infection (Fig. 6).

The major advantages of functionalized TiO_2_ NPs are their effectiveness at lower concentrations, the low cost of their synthesis, molecular specificity, and ease in designing unique adsorbed with flavonoids and terpenes (Gurunathan et al. 2020). Therefore, functionalized TiO_2_ NPs exhibit strong potential to be developed as anti-SARS-CoV-2 therapy. Taken together, our findings support the model by which FTNP prevents virus-cell interaction, which are key steps for successful viral infection of the host cells, and therefore, NPs-based compounds present a useful therapeutic approach to prevent SARS-CoV-2 entry and cell-to-cell spread.

In conclusion, our molecular docking studies showed that the flavonoids H7R and F7G bind with high affinity to specific sites in the SARS-CoV-2 spike, which are involved in the transition to the open configuration that is required for binding to the ACE-2 receptor. Furthermore, in parallel, the molecular dynamics simulations indicate that H7R and F7G form a stable complex with the proposed binding site on SARS-CoV-2 spike. Although both molecular docking and molecular dynamics were performed with flavonoids not adsorbed to TiO_2_, in vitro activity showed that their presence, enhances their antiviral activity.

The antiviral activity of FTNP was finally evaluated utilizing a multidisciplinary experimental approach. Our results demonstrated the potent antiviral activity in vitro against two different coronaviruses: CHoV-229E and SARS-CoV-2 under in vitro model. The mechanism of this effect would involve a blockade of the viral infective process. Cell fusion experiments demonstrated that FTNP (specifically the flavonoids H7R and F7G) effectively prevent the spike protein binding to the ACE-2 receptor. This mechanism provides a plausible mechanism for the antiviral effects of FTNP against SARS-CoV-2. Finally, it is important to note that this study is part of a project authorized by the Research Ethics and Investigation Committee of the “Mónica Pretelini Sáenz” Maternal-Perinatal Hospital (HMPMPS) (code 2020–07-691), with current registration with the National Bioethics Commission (CONBIOETICA) as well as by the Research Committee of the same Hospital with current registration in the Federal Commission for the Protection against Health Risks (COFEPRIS 213300410A0034/2021). At this point, a clinical trial protocol is being formulated to determine the FTNP effectiveness to treat COVID-19 human patients.

Supplementary information.

## Supplementary Information

Below is the link to the electronic supplementary material.

Supplementary file1 (PDF 822 KB)

## Data Availability

Molecular dynamic data, mass spectroscopy, X-ray diffraction, and transmission electron microscopy are available as supplementary material. GLG is the author of 3 approved patents (MX Patent 339086, JP Patent 6625051, US. Patent US10342840B2). These patents are cited in this work and describe how functionalized nanoparticles can be obtained.
