# Supplementary material for: A potent virucidal activity of functionalized TiO2 nanoparticles adsorbed with flavonoids against SARS-CoV-2
Source: Appl Microbiol Biotechnol. 2022 Aug 11;106(18):5987–6002. doi: 10.1007/s00253-022-12112-9 (PMC9366830; doi:10.1007/s00253-022-12112-9)
Supplement: Supplementary file 1 — Supplementary file1 (PDF 822 KB) [file 253_2022_12112_MOESM1_ESM.pdf]

A potent virucidal activity of TiO_2 functionalized nanoparticles adsorbed with flavonoids against SARS-CoV-2

Applied Microbiology and Biotechnology

Gabriela León-Gutiérrez[1], James Edward Elste[2], Carlos Cabello-Gutiérrez[3], Cesar Millán-Pacheco[4], Mario H. Martínez-Gómez[1], Rafael Mejía-Alvarez[5], Vaibhav Tiwari[2] and Armando Mejía[1]*

[1]Departmento de Biotecnología, Universidad Autónoma Metropolitana–Iztapalapa, Ciudad de México, México.

[2]Department of Microbiology and Immunology, Midwestern University, Downers Grove, IL, USA

[3]Departamento de Virología e Investigación en Micología, Instituto Nacional de Enfermedades Respiratorias, Ciudad de México, México

[4]Facultad de Farmacia, Universidad Autónoma del Estado de Morelos, Cuernavaca, Morelos, México

[5]Department of Physiology, College of Graduate Studies, Midwestern University, Downers Grove, IL, USA.

*Corresponding author: **ama@xanum.uam.mx**

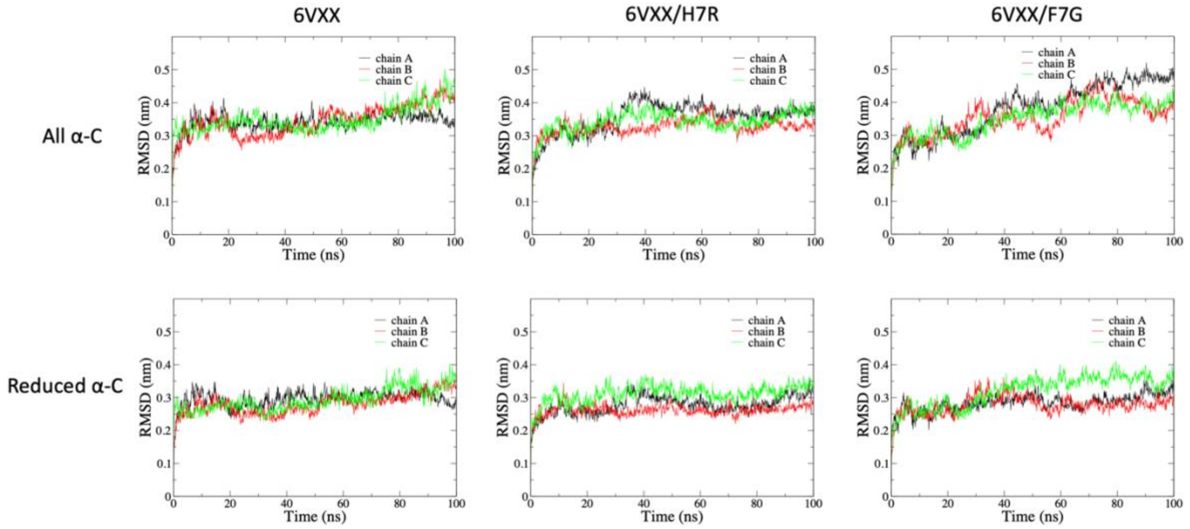

Figure S1 α-Carbon root mean square deviation (RMSD) along time. α-Carbon of first and last 15 residues (amino and carboxyl terminal) and loop from 676 to 689 was not consider for the RMSD calculation on the reduced α-carbon graphs

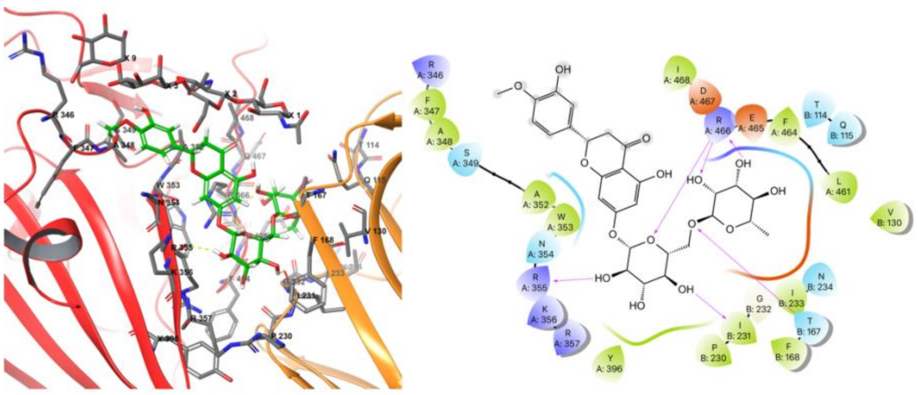

6VXX/H7R

Figure S2 Three-dimensional interaction maps for SARS-CoV2 spike glycoprotein and interacting H7R. H7R is shown with carbons on green. All residues at least 5Å are shown on both representations. Sugars are named as X1, X2, X3, X9 and X13 and not shown on the interaction map. Hydrogen bonds are shown as dotted lines yellow on the three-dimensional representation and as pink arrows on the interaction maps

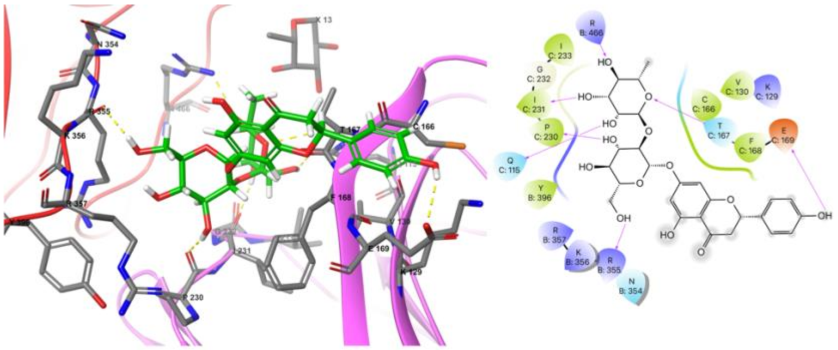

6VXX/F7G

Figure S3 Three-dimensional and interaction maps for spike glycoprotein and interacting F7G. F7G is shown with carbons on green. All residues at least 5Å are shown on both representations. Sugars are named as X1, X2, X3, X9 and X13 and not shown on the interaction map. Hydrogen bonds are shown as dotted lines yellow on the three-dimensional representation and as pink arrows on the interaction maps

Figure S4 a. X-ray diffraction peak of TiO2 NPs. XRD measurements show that TiO_2 NPs exhibit the anatase structure. The average grain size calculated from the broadening of the XRD peak of anatase was 2 nm.
b. Transmission electron microscope (TEM) image of anatase TiO2 NPs. TEM showed the average size of nanoparticles suspended of 2 nm, consistent with the XRD results

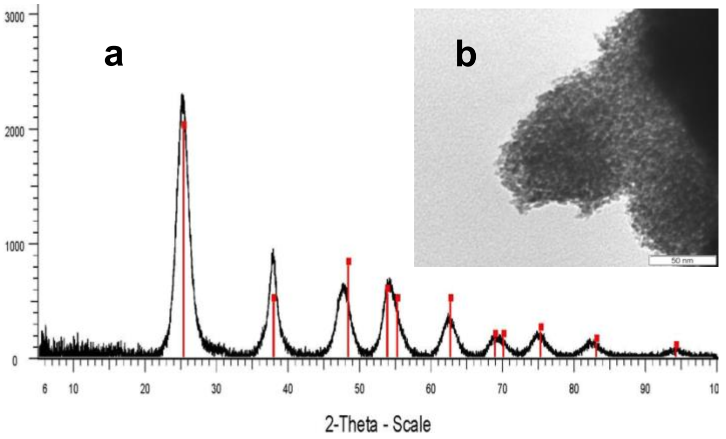

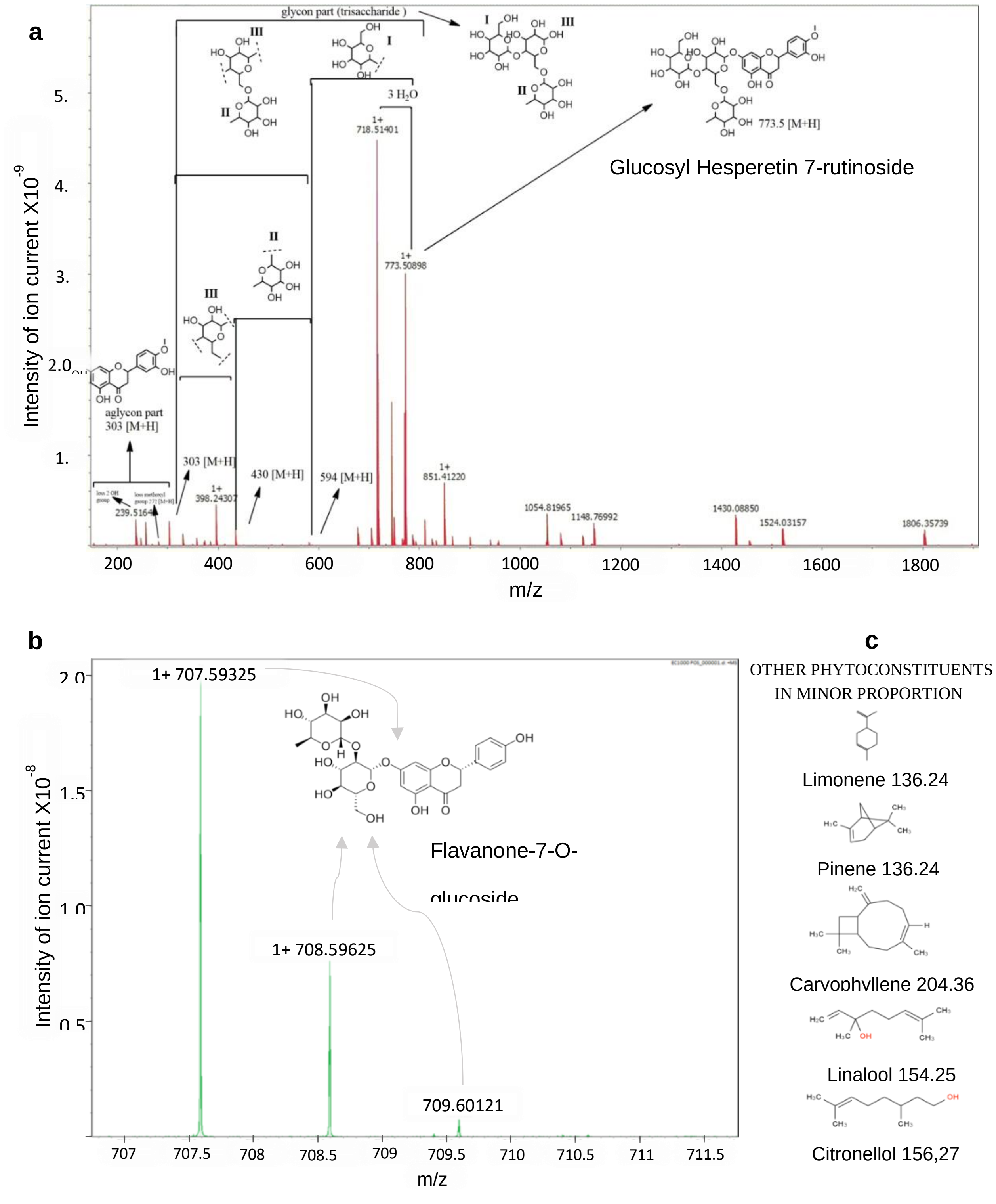

Figure S5. ESI FT-ICR. **a.** Representative positive ion mode mass spectra of (H7R) Glycosil-Hesperetin (isotopic pattern: 773.50, 774.51, 775.51). **b.** Representative negative ion mode mass spectra of (F7G) Naringin -H++ 65Cu 2++ 2CH3OH (isotopic pattern: 707.59, 708.59, 709.60). **c.** Representative positive ion mode mass spectra of terpenes

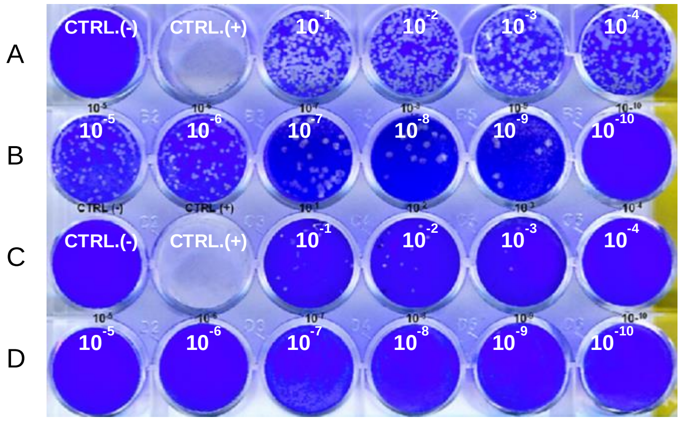

Figure S6 Plaque assay virus titration. **Lane A and B.** Coronavirus ChoV-229E in VERO.E6 cells as control. PFU/mL= $6x10^{10}$. **Lane C and D.** Coronavirus 229E pre-incubated for 5 minutes with F7G. PFU/mL= $3x10^{4}$

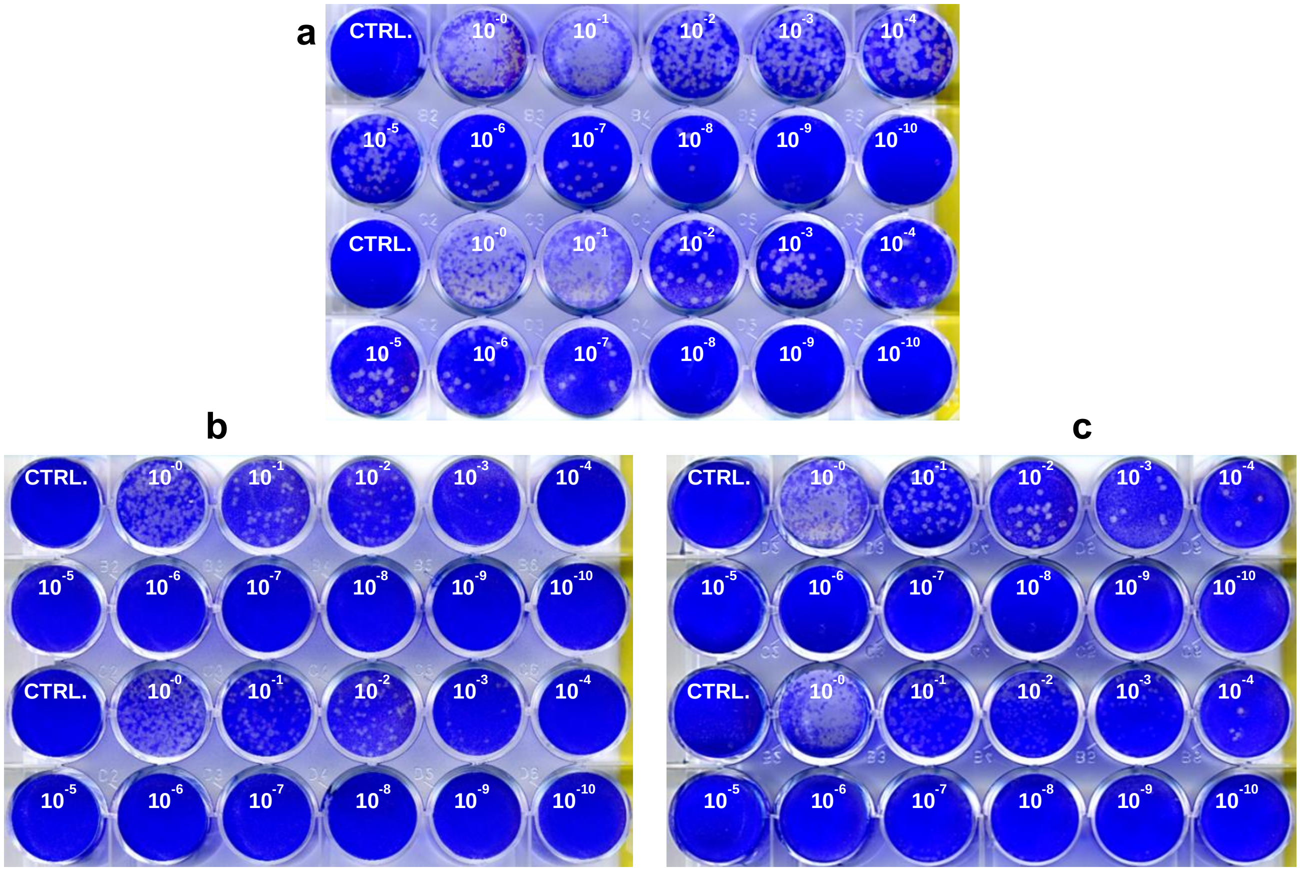

Figure S7 Plaque assay virus titration. **a.** SARS-CoV-2 in VERO.E6 cells as control. PFU/mL= $3x10^{9}$ **b.** SARS-CoV-2 pre-incubated for 5 minutes with F7G. PFU/mL= $5x10^{4}$ **c.** SARS-CoV-2 pre-incubated for 5 minutes with H7R. PFU/mL= $3x10^{5}$
